# Effects of Physiological Levels of the Green Tea Extract Epigallocatechin-3-Gallate on Breast Cancer Cells

**DOI:** 10.3389/fendo.2014.00061

**Published:** 2014-05-07

**Authors:** Li Zeng, Jeff M. P. Holly, Claire M. Perks

**Affiliations:** ^1^IGFs and Metabolic Endocrinology Group, School of Clinical Sciences, Southmead Hospital, University of Bristol, Bristol, UK

**Keywords:** EGCG, breast cancer, physiological, green tea, drug sensitivity

## Abstract

Physiological concentrations of the green tea extract epigallocatechin-3-gallate (EGCG) caused growth inhibition in estrogen receptor α (ERα)-positive MCF7 cells that was associated with down-regulation of the ERα and reduced insulin-like growth factor binding protein-2 abundance and increased protein abundance of the tumor suppressor genes p53/p21. In contrast to MCF7 cells that have wt p53, EGCG alone did not change cell proliferation or death significantly in another ERα-positive cell line T47D that possesses mutant p53. EGCG increased ERα protein levels and as a consequence, the cells responded significantly better to an ERα antagonist tamoxifen (TAM) in the presence of EGCG. EGCG significantly increased cell death in an ERα-negative cell line, MDA-MB-231 that also possesses mutant p53. EGCG significantly increased the ERα and insulin-like growth factor-I receptor levels and thereby enhanced the sensitivities of the cells to TAM and a blocking antibody targeting the insulin-like growth factor-1 receptor (αIR3). In contrast to MCF7, T47D and MDA-MB-231 breast cancer cells that exhibited significant changes in key molecules involved in breast growth and survival upon treatment with physiological levels of EGCG, the growth, survival, and levels of these proteins in non-malignant breast epithelial cells, MCF10A cells, were not affected.

## Introduction

Tea originated from China and has been produced and consumed for thousands of years. Due to different manufacturing methods, tea is produced in different forms. Green tea is made from fresh tea leaves (*Camellia sinensis*). Tea is the most widely consumed beverage next to water and provides a source of the well-known polyphenols, which are associated with a reduction in cancer risk (1). After steaming or pan-frying, enzymes are inactivated to prevent the oxidation of tea polyphenols, which are also called catechins. Catechins account for 30–40% of the dry weight of the solids in brewed green tea. There are four major catechins in green tea: (−)-epigallocatechin-3-gallate (EGCG), (−)-epigallocatechin (EGC), (−)-epicatechin gallate (ECG), and (−)-epicatechin (EC) (2). EGCG is the most abundant and biologically active polyphenolic catechin in green tea, and exerts multiple effects in humans. A variety of laboratory experiments, animal models, and epidemiology studies indicate the protective effects of many dietary agents against tumorigenesis, including EGCG (3). While the cancer preventive effects of green tea have been well established in animal models, its activity in humans is still controversial (4).

Breast cancer is now the most common cancer in developed countries. Despite decreased mortality due to improved prevention, detection by use of screening mammography and therapy options including endocrine therapy, incidence of breast cancer is still increasing. About one in eight (12%) women in the US will develop invasive breast cancer during their lifetime (American Cancer Society^1^). In the last 10 years, female breast cancer incidence rates in the UK have increased by 6% (Cancer Research UK). Even in countries that used to have lower incidences of breast cancer, such as Japan and China, have observed increases in breast cancer incidence due to the adoption of a more westernized life style (5).

The major issue with the majority of research studying the effects of EGCG is that the levels of EGCG are super-physiological (from 20 to 200 μM) and such concentrations cause cytotoxic effects to normal cells, potentially causing unwanted side effects. A physiological serum concentration of EGCG (<10 μM) can be achieved by drinking a couple of cups of green tea or taking a tablet supplement (6, 7), and the effects of these doses have not been well investigated.

Among many other cancers, EGCG has been found to inhibit cancer development in lung (8) (10–40 μM EGCG) (9) (262 μM EGCG), prostate (10) (20–80 μM EGCG), colon (11) (20 μM EGCG), skin (12) (21–87 μM EGCG), and breast cancers (13) (87–131 μM EGCG). A variety of mechanisms have been proposed as to how EGCG imparts its chemo-preventive effects, including inhibition of MAP-kinase, AP-1 (14), NFκB, angiogenesis, invasiveness, metastasis (15), and DNA methyl-transferase (DNMT) (16); induction of apoptosis; modulation of cell cycle checkpoint controls (8); transcription factor expression; and receptor-mediated functions (17). A recent study showed that with MCF7 and MDA-MB-231 cells, EGCG and a pro-drug of EGCG (pEGCG, EGCG octaacetate) caused hypomethylation of human telomerase reverse transcriptase (hTERT) gene via inhibition of histone deacetylase (HDAC) and histone acetyltransferase (HAT) activity. Demethylation of hTERT established a transcription repressing environment to prevent aberrant hTERT expression and lead to tumor suppression (18). pEGCG was synthesized by modulation of hydroxyl groups with peracetate groups to enhance the bioavailability and stability of EGCG. The same group also reported that combining EGCG and a HDAC inhibitor trichostatin (TSA) synergistically re-activated a functional estrogen receptor in MDA-MB-231 cells via altering the binding transcription repressor complex pRb2/p130–E2F4/5–HDAC–DNMT1–SUV39H1 to the estrogen receptor α (ERα) promoter. This induction of ERα expression could sensitize ERα-negative breast cancers to anti-hormone therapy (19).

In this study, we aimed to assess if physiological concentrations of EGCG affected cell growth, cell death, and altered key molecules [insulin-like growth factor-1 receptor (IGF-1R), ER, and HER2] that have been implicated in regulating these processes and if such changes influenced the sensitivity to agents targeting breast cancer cells.

## Materials and Methods

All chemicals were purchased from Sigma (Gillingham, Dorset, UK) unless otherwise stated. αIR3 was bought from Calbiochem, Nottingham, UK, and herceptin was a kind gift from AstraZeneca, Cheshire, UK.

### Cell culture

The estrogen receptor negative human breast cancer cell line MDA-MB-231 was purchased from ECACC. The estrogen receptor positive human breast cancer cell lines MCF7 and T47D and the relatively normal breast epithelial cell line MCF10A were obtained from ATCC. Cells were maintained in growth media (GM) at 37°C and 5% CO_2_ in a humidified incubator. Growth medium for MCF10A consisted of a 1:1 mixture of Ham’s F12 medium and Dulbecco’s modified Eagle’s medium with 2.5 mM l-glutamine (DMEM:F12, Gibco, Paisley, UK), 5% horse serum (Gibco, Paisley, UK), 20 ng/ml EGF (Calbiochem, Nottingham, UK), 100 ng/ml cholera toxin, 10 μg/ml insulin (Novo Nordisk, West Sussex, UK), and 0.5 μg/ml hydrocortisone. MCF7, T47D, and MDA-MB-231 cells were cultured in DMEM supplemented with 10% fetal bovine serum (FBS). All GM contain penicillin (50 IU/ml), streptomycin (50 IU/ml), and l-glutamine (2 mM). Experiments were performed in serum-free media (SFM) [DMEM:HamsF12 supplemented with sodium bicarbonate (0.12%), BSA (0.02%), apo-transferrin (0.1 mg/ml), penicillin (50 IU/ml), streptomycin (50 IU/ml), and l-glutamine (2 mM)]. Cells were seeded onto 6- or 24-well plates in GM and transferred to SFM 24 h later. Dosing was performed after 24 h in SFM. Cells were placed into fresh SFM and treated as detailed in the figure legends.

### Cell counting

Both attached and floating cells were collected and prepared for counting using a hemocytometer. Cells were mixed with trypan blue dye to distinguish live and dead cells. Cells were counted from which total cell number and the percentage of dead cells relative to control were calculated.

### Tritiated thymidine incorporation

Proliferation was also measured using [3H]-thymidine incorporation. 0.1 μCi of [^3^H]-thymidine (Perkin Elmer Beaconsfield, Bucks, UK) was added to the cells for the last 4 h of treatment. Cells were then washed in 5% trichloroacetic acid (TCA) for 10 min at 4°C, followed by lysing in 1 M sodium hydroxide for 1 h at room temperature. Lysates were mixed with ultima gold liquid scintillation cocktail (Perkin Elmer Beaconsfield, Bucks, UK) and incorporated counts were measured using a Beckman Scintillation Counter LS6500. Data were recorded as disintegrations per minute (DPM).

### Western blotting

Cell lysates and media were run on 12% SDS-PAGE gel and proteins transferred to a Hybond-C nitrocellulose membrane (GE Healthcare, Bucks, UK). Proteins were probed with anti-insulin-like growth factor binding protein-2 (IGFBP-2) 1:1000 (sc-6001 Santa Cruz); anti-ERα 1:750 (sc-73479 Santa Cruz, TX, USA); anti-PARP 1:1000 (556494 BD, Oxford, UK); anti-GAPDH 1:5000 (MAB 374 Millipore, Darmstadt, Germany); anti-α-tubulin 1:5000 (ABJ1178 Autogen Bioclear, Wiltshire, UK); anti-Her2 1:1000 (#2248 Cell Signaling, Hertfordshire, UK); anti-IGF-I receptor (IGF-IR) 1:1000 (D23H3 Cell Signaling, Hertfordshire, UK); anti-p53 1:1000 (sc-126 Santa Cruz, TX, USA); anti-p21 1:2000 (05-345 Upstate Biotechnology, New York, NY, USA); or anti-β-actin 1:10000 (A5441 Sigma-Aldrich, Gillingham, Dorset, UK) following the manufacturer’s instructions. Secondary antibodies were diluted in 5% milk-TBST (20 mM Tris, 136 mM sodium chloride, 0.1% Tween-20, pH 7.4) and proteins visualized using supersignal west dura ECL solution (Thermo Fischer, Ulm, Germany) and the UVP Chemi-Doc-IT imaging system (Bio-Rad, Hertfordshire, UK), as described previously (20).

### RIA

IGF-II was measured in MDA-MB-231 cell conditioned media by RIA as described previously (21).

### Statistical analysis

The data were analyzed with SPSS 12.0.1 for Windows using one-way ANOVA followed by least significant difference (LSD) *post hoc* test. A statistically significant difference was considered to be at *p* < 0.05.

## Results

### EGCG at physiological concentrations inhibited cell proliferation and increased cell death of breast cancer cells

**Figure 1:**
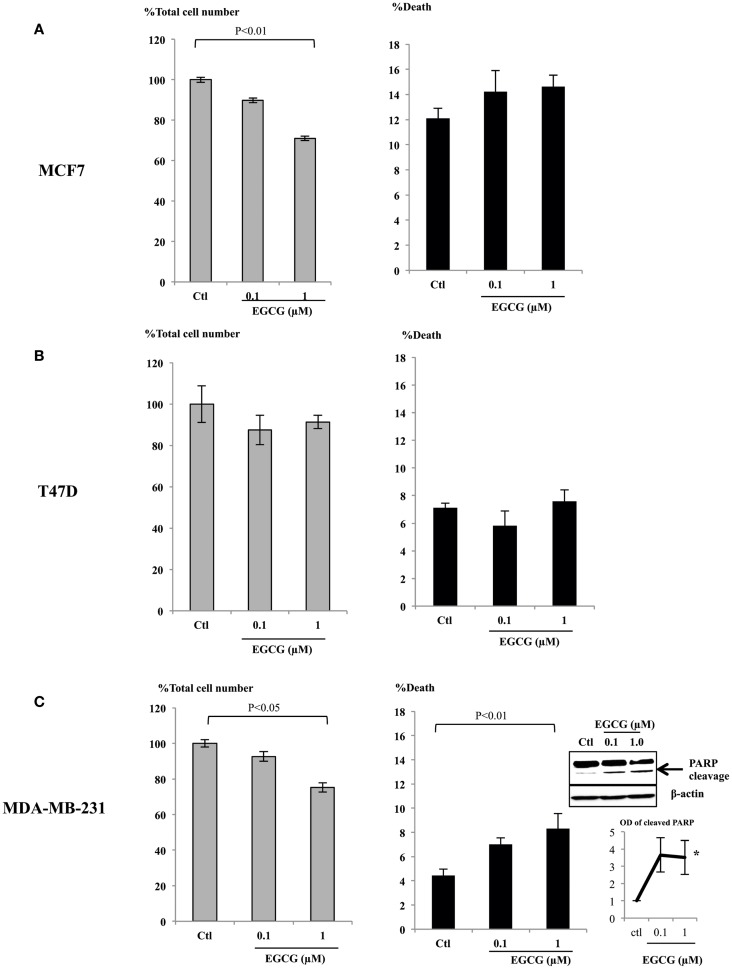
**MCF7 (A), T47D (B), and MDA-MB-231 (C) cells were seeded (0.2 × 10^6^) in six-well plates in GM and after 24 h in SFM were dosed with EGCG (0–1 μM) for 48 h**. Graphs show percentage of total cell numbers compared to the untreated control (left panel) and percentage of cell death (right panel) assessed by trypan blue exclusive cell counting. Graphs are means from at least three independent repeats, each in triplicate upon which statistical analysis was performed. Insert shown in **(C)** is a western blot showing an increase in PARP cleavage together with a graph showing the mean OD measurements of blots from three separate experiments.

It has been reported that physiological, achievable serum concentration of EGCG is not higher than 1 μM (22–24) or up to 7 μM with a supplement (25). To analyze whether these physiological levels of EGCG have any impact on breast cancer cell proliferation, we assessed doses of EGCG up to 1 μM in ERα-positive breast cancer cell lines, MCF7 (Figure 1A), T47D (Figure 1B), and an ERα-negative cell line MDA-MB-231 (Figure 1C). The percentages of total cell number compared to the control samples are shown. With 1 μM EGCG, growth inhibition was observed in MCF7 (28%, *p* < 0.01) and MDA-MB-231 (25%, *p* < 0.05) cells, but cell growth was not significantly affected in T47D (8%) cells. While no significant increase in cell death was achieved with 1 μM EGCG in MCF7 or T47D cells, EGCG triggered a doubling in cell death (*p* < 0.01) in MDA-MB-231 cells, compared to untreated cells. We confirmed this was apoptotic cell death by showing an increase in PARP cleavage at 0.1 and 1 μM (insert Figure 1C).

### Physiological concentrations of EGCG increased ERα and IGF-IR abundance in MDA-MB-231 cells and sensitized them to tamoxifen and an IGF-IR inhibitor (αIR3)

**Figure 2:**
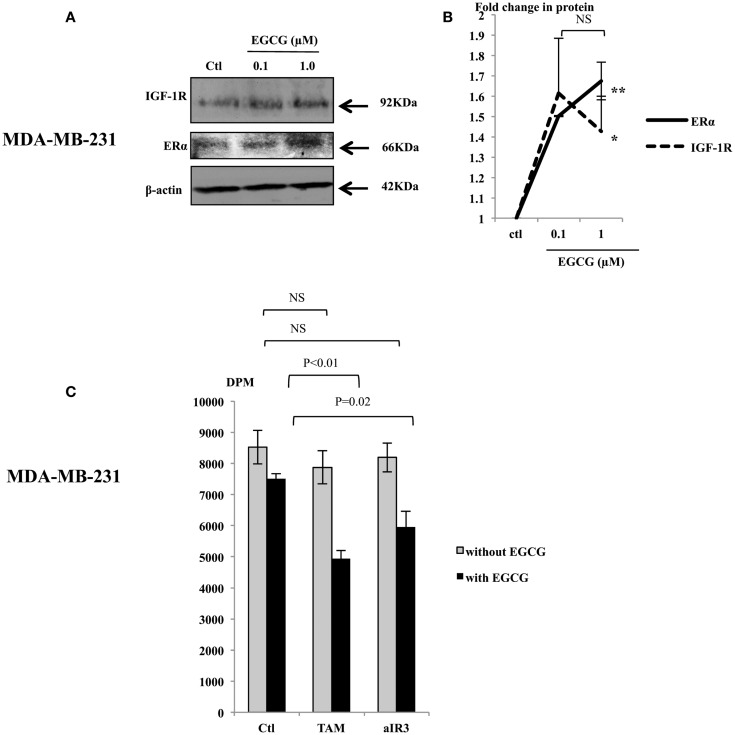
**Representative western immunoblots showing abundance of IGF-1R and ERα in MDA-MB-231 cells with whole cell lysates (100 μg) following EGCG treatment (0–1 μM) for 48 h (A), β-actin was assessed to show equal loading of the protein**. Fold changes of the proteins were shown by densitometry measurements **(B)**, which are mean value of at least three repeats. MDA-MB-231 cells were seeded and treated similarly with EGCG. Tamoxifen (TAM, 1 μM) or αIR3 (1 μg/ml) were dosed to the cells 48 h after EGCG treatment. DNA synthesis was measured using TTI assay after 48 h of TAM/αIR3 treatment **(C)**. Graphs show the mean value of DPM from at least three experiments each performed in triplicate upon which statistical analysis was performed; *p < 0.05, **p < 0.01.

In order to further understand the effects of EGCG in MDA-MB-231 cells, we assessed changes in the abundance of the IGF-IR and the ERα following treatment with EGCG. EGCG (1 μM) caused an increase in their expression (Figure 2A): a 1.42 (*p* < 0.05) and 1.67 (*p* < 0.005) fold increase, respectively, compared to untreated controls (Figure 2B). We also observed that levels of HER2 were undetected and unaffected following treatment with EGCG (data not shown). We also found that the MDA-MB-231 cells secreted approximately 30 ng/ml IGF-II as measured by RIA.

We then tested the sensitivity of MDA-MB-231 cells to TAM and αIR3, which blocks ERα and IGF-IR pathways, respectively (Figure 2C). Initial experiments looking at the effects of EGCG were examining changes in cell number and cell death and therefore we used cell counting. In addressing the effects on the response to TAM and αIR3, as these affect growth but do not induce apoptosis at the doses used, we used thymidine incorporation as a more sensitive measure of changes in cell proliferation. Due to low level of the ERα and IGF-IR basally, as anticipated, MDA-MB-231 cells did not respond to TAM or αIR3 in terms of cell proliferation. But with pre-treatment of 1 μM EGCG, TAM and αIR3 inhibited cell growth by 34% (*p* < 0.01) and 21% (*p* = 0.02), respectively.

### Treatment with EGCG increased the protein abundance of ERα, Her2, and IGFBP-2 in T47D cells and sensitized them to tamoxifen, but not to herceptin

**Figure 3:**
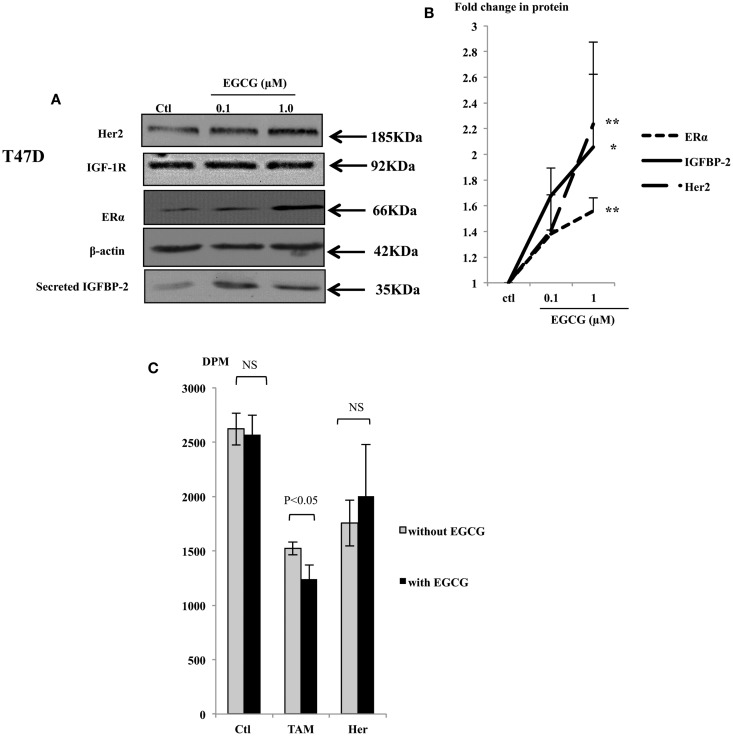
**Western immunoblot showing abundance of Her2, IGF-1R, and ERα from 50 μg whole lysates of T47D and secreted IGFBP-2 in the supernatants (A), following EGCG treatment (0–1 μM) for 48 h**. β-actin was assessed to show equal loading of the protein. IGFBP-2 secretion was assessed with 30 μl un-concentrated supernatant. They are representative blots of experiments repeated at least three times. Fold changes of the proteins were shown by densitometry measurements **(B)**. Sensitivity of the T47D cells to tamoxifen or herceptin **(C)** was determined by seeding cells (0.025 × 10^6^) in 24-well plates in GM 24 h before they were placed into SFM for a further 24 h, then treated with 1 μM EGCG. One micromolar tamoxifen (TAM) or 10 μg/ml herceptin (Her) were dosed to cells at 48 h after EGCG treatment. DNA synthesis was measured using tritiated thymidine incorporation assay after 48 h of TAM/Her treatment. Graphs show the mean value of DPM from at least three experiments each performed in triplicate upon which statistical analysis was performed; *p < 0.05, **p < 0.01.

With T47D cells, EGCG at the physiological concentrations increased the abundance of ERα, Her2, and IGFBP-2 protein (Figure 3A), but the abundance of IGF-IR protein was not affected (Figure 3A). The ERα, Her2, and IGFBP-2 expression was increased with 1 μM EGCG by 1.6 (*p* < 0.001), 2.23 (*p* < 0.02), and 2.06 (*p* < 0.05) fold, respectively (Figure 3B).

As shown in Figure 1, while low concentrations of EGCG alone caused growth inhibition in the MCF7 cells, it had little effect in T47D cells. Compared to MCF7 cells, T47D express lower levels of the ERα and are less responsive to TAM treatment. With low expression of Her2, monoclonal antibodies targeting Her2, such as herceptin, are also not particularly effective in blocking cell proliferation in these cells. As an increased expression of the ERα and Her2 was observed in T47D cells in response to EGCG, we further examined whether the sensitivity of these cells to TAM and herceptin could be improved when they were combined with 1 μM EGCG.

Tamoxifen alone inhibited cell growth in T47D cells by 42%, 1 μM of EGCG did not cause significant growth inhibition in these cells as we saw previously, but combining both together gave a 52% decrease in cell growth, which was higher than each of them separately (*p* < 0.05) (Figure 3C). This implies that in T47D cells, EGCG synergistically enhanced their sensitivity to TAM probably due to elevated ERα expression. Although T47D cells express relatively low levels of the Her2 receptor, they still responded to herceptin with 28 and 23% inhibition of cell growth with or without EGCG treatment, respectively, which was not significantly changed.

### Treatment with EGCG changed the expression of key proteins involved in cell growth in MCF7 cells

**Figure 4:**
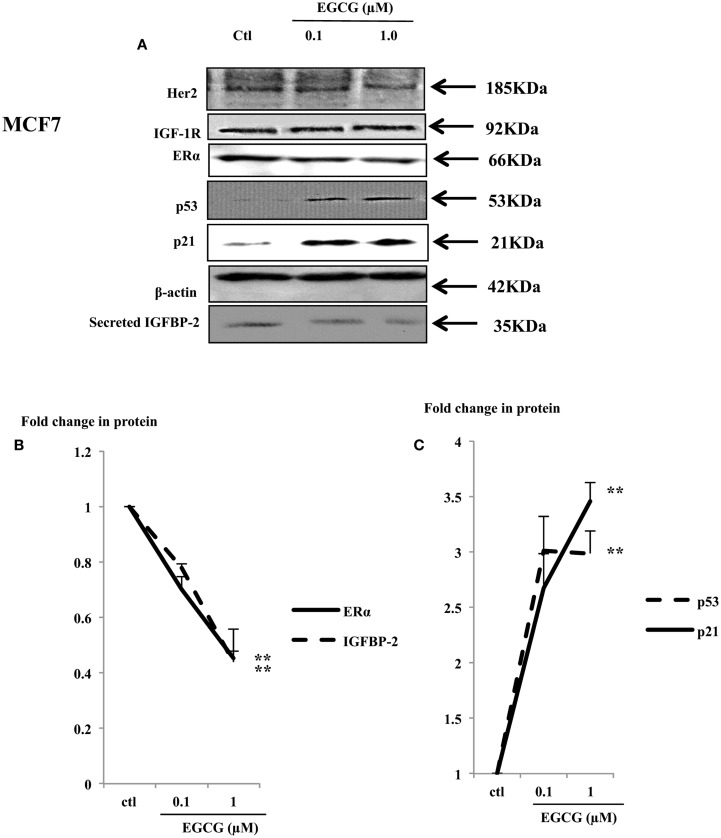
**Western immunoblot showing abundance of ERα, p53, and p21 in whole lysates of MCF7 (50 μg) following EGCG treatment (0–1 μM) for 48 h (A)**. β-actin was assessed to show equal loading of the protein. IGFBP-2 secretion was assessed with 30 μl un-concentrated supernatant. They are representative blots of experiments repeated at least three times. Fold changes of these proteins were shown by densitometry measurements **(B,C)**; *p < 0.05, **p < 0.01.

Physiological concentrations of EGCG decreased cell proliferation in MCF7 cells (Figure 1A). Her2 and IGF-1R were not changed (Figure 4A), but the ERα and IGFBP-2 abundance decreased to 45% (*p* < 0.002) and 44% (*p* = 0.02) of the untreated control, respectively (Figures 4A,B).

The tumor suppressor gene p53 is mutated in T47D and MDA-MB-231 cells and has lost its function (26, 27). In contrast MCF7 cells possess wild-type P53 which acts as a tumor suppressor gene by playing a role in maintaining genetic integrity (28). A dose-dependent increase in p53 and its downstream effector p21 was observed (Figure 4A) with a 3 (*p* < 0.001) and 3.5 (*p* < 0.02) fold increase with 1 μM EGCG, respectively (Figure 4C).

### EGCG at physiological concentrations had no effects on the normal breast epithelial cells

**Figure 5:**
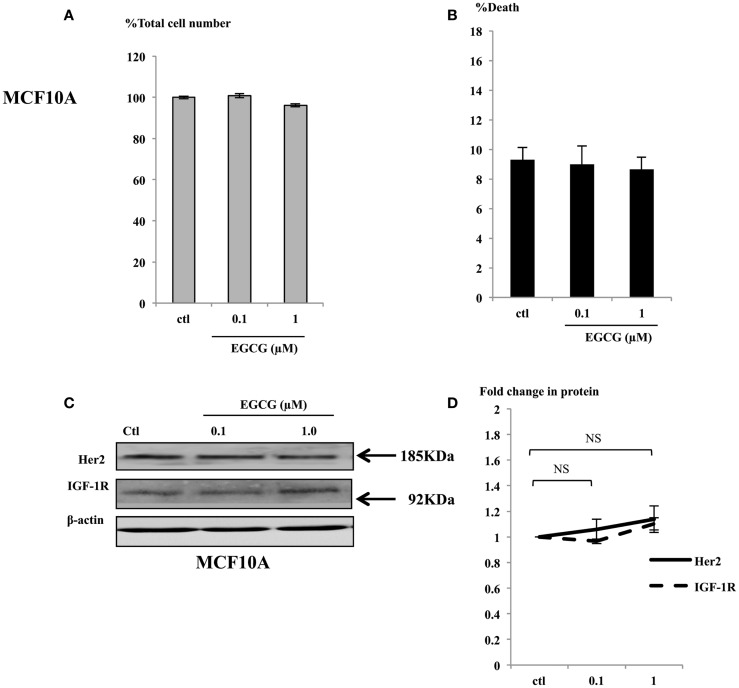
**MCF10A cells were seeded (0.2 × 10^6^) in six-well plates in GM and after 24 h in SFM were dosed with EGCG (0–1 μM) for 48 h**. Graphs show percentage of total cell numbers compared to the untreated control **(A)** and percentage of cell death **(B)** assessed by trypan blue exclusive cell counting. Graphs are means from at least three independent repeats, each in triplicates. Western immunoblot showing abundance of Her2 and IGF-1R in MCF10A cells with whole cell lysates (100 μg) following EGCG treatment (0–1 μM) for 48 h **(C)**. β-actin was assessed to show equal loading of the protein. They are representative blots of experiments repeated at least three times. Fold changes of these proteins were shown by densitometry measurements **(D)**.

In contrast to the effects seen in the cancer cells exposed to physiological concentrations (up to 1 μM), the MCF10A cells showed no differences in cell growth (Figure 5A) or induction of cell death (Figure 5B). Consistent with the phenotype observed in the non-malignant MCF10A breast cells, the expression of the key proteins involved in breast cell proliferation, such as IGF-1R and Her2 were analyzed in whole cell lysates of MCF10A cells treated with EGCG and were found not to change (Figures 5C,D). Other key proteins such as the ERα and IGFBP-2 were also unchanged and p53 and p21 were undetectable (data not shown).

## Discussion

For thousands of years, green tea has been known to exert health-promoting effects in various conditions: cancer, cardiovascular diseases, neuro-degenerative diseases, strokes, obesity, diabetes, and many viral or bacterial infections. The most abundant catechin in green tea is EGCG. Its bioactivity, stability, potential side effects, and usage in clinical trials have been widely discussed (3, 25, 29). However the *in vitro* studies that endeavor to mimic *in vivo* effects generally used EGCG at concentrations higher than 10 μM and even as high as 200 μM, which are physically un-achievable in the human body. Cancer-specific toxicity is a crucial element in breast cancer therapy. Many anti-cancer drugs used in the clinic are limited by their general toxic side effects (30). Physiological concentrations of EGCG in human plasma reach between 0.1 and 1 μM, and may approach 7 μM with supplements. In order to study whether and how EGCG at a physiological achievable concentration may potentially be beneficial to breast cancer patients, we used a range of 0.1–1 μM EGCG to assess its actions on breast cancer cells. The non-malignant breast epithelial cell line MCF10A was also used as a control to examine the cancer-specificity of EGCG.

The most exciting finding from this work is that physiological concentrations of EGCG exerted cancer-selective growth inhibitory and pro-apoptotic effects. It also altered the expression of many key proteins involved in cancer growth and survival, with no effect on these molecules in normal cells. This in turn enhanced the sensitivity of cancer cells to current therapies. Although TAM has been successfully used in ERα-positive breast cancers, about 30% of patients are ERα- and/or progesterone receptor (PR)-negative and resistant to endocrine modification and therefore display poor prognosis. In addition, a proportion of hormone positive cancers that initially respond to hormone therapy eventually develop hormone resistance and become more aggressive. If a cancer also lacks Her2 expression, they are described as being triple negative (TNBC). MDA-MB-231 is an example of a TNBC cell line which lacks ERα, PR, and Her2 expression and is resistant to hormone therapy.

With MDA-MB-231, we found the induction of cell death was a dominant consequence of EGCG treatment by itself. In addition, EGCG also increased ERα abundance in these cells and as a result of this, the cells were then able to respond to TAM.

Chrisholm et al. also showed cytotoxic effects of EGCG alone in another ERα-negative breast cancer cell line, Hs578T and a synergistic cytotoxic effect of EGCG with TAM in MDA-MB-231 cells (31), but at much higher, non-physiological concentrations.

Various studies using EGCG found that it regulated tumor suppressor genes through DNA demethylation (32, 33) or histone re-acetylation in skin (34), breast (35), prostate (36), colon, and esophageal cancer (37). In the ERα-negative MDA-MB-231 cells, it was reported that EGCG re-activated ERα expression at 10 μM and synergistically regulated ERα re-expression with AZA and TSA (19). The modulation of the chromatin markers including acetyl-H3, acetyl-H3K9, acetyl-H4, dimethyl-H3K4, and trimethyl-H3K9 indicated epigenetic regulation by EGCG in MDA-MB-231 cells. It is also suggested that histone modification mechanisms may play a more important role in EGCG-induced-ERα reactivation than DNA methylation in ERα-negative breast cancer cells. Our data also show that EGCG re-expressed the ERα but at physiological concentrations. Examining if this is by the same epigenetic mechanism would be interesting as this would more easily be translated into the clinic. In addition, we found that the MDA-MB-231 cells were still unable to respond to exogenous estradiol despite re-expression of the ERα (data not shown).

Unlike the data from Chrisholm et al., who did not observe growth inhibitory effects of EGCG in ERα-positive breast cancer cells (31), we found EGCG alone at physiological levels did have inhibitory actions on cell growth in MCF7 cells. The tumor suppressor gene p53 is mutated in T47D and MDA-MB-231 cells and has lost its function (26, 27). But wild-type p53 is present in MCF7 cells and acts as a tumor suppressor gene by playing a role in maintaining genetic integrity (28). A dose-dependent decrease in ERα abundance together with an increase in p53 and p21 in response to EGCG may contribute to the decreased cell proliferation. These results are consistent with a report from Liang et al. (38), in which 30 μM EGCG caused an accumulation of p53, p21, and p27 in MCF7 cells, which was purported to contribute to EGCG-induced cell cycle G1 arrest. Our new data suggest that even very low, physiological concentrations of EGCG can simulate changes in abundance of key anti-proliferative proteins that leads to inhibition of cell growth. Very recently, an EGCG-induced decease of ERα transcription and expression in ERα-positive breast cancer cells MCF7 and T47D at the promoter activity level has been reported (39). However, non-physiological concentrations of EGCG were used (20 μM and above). It will be interesting to investigate if the same mechanism underlies the changes of ERα protein expression in MCF7 observed in our study using achievable concentrations of EGCG. We and others have found that the demethylating agent AZA induced a similar down-regulation of ERα in the ERα-positive breast cancer cell lines MCF7 and T47D, but not via epigenetic modulation (40, 41).

Using physiologically doses with T47D cells, we found that in contrast to MCF7 cells, EGCG actually caused an increase in abundance of the ERα. In these cells, the growth inhibition was unaffected by low doses of EGCG, but having observed that EGCG increased the ERα abundance, we combined treatment of EGCG with TAM, which targets ERα and observed an additive growth inhibition but reassuringly the increase in the ERα was not accompanied by an enhanced proliferative response to estradiol (data not shown).

Although ERα is the main driver of breast cancer progression and still the main target for treatment, dysregulation of the IGF-1R/phosphatidylinositol-3-kinase (PI3K)/Akt pathway has been shown to correlate with breast cancer development and has been intensively studied as a potential therapeutic target (42–44). The trans-membrane receptor IGF-IR is a tyrosine kinase receptor and mediates insulin-like growth factor (IGF) activities. Increased levels of the IGF-IR have been implicated in many cancers including breast (42) and prostate cancer (45). IGF-IR signaling stimulates cell growth and inhibits death (46). Among different potential approaches to treat TNBC, some small molecular inhibitors or neutralizing antibodies targeting IGF-IR have been designed to block IGF-IR pathway and therefore to reduce cancer cell growth. αIR3 is a monoclonal antibody that acts as an IGF-IR antagonist (47). Blockade of tumor growth *in vivo* and *in vitro* has been observed with treatment of αIR3 in MDA-MB-231 cells (48). We have shown here that with MDA-MB-231 cells, physiological concentrations of EGCG increase the IGF-IR and improve their response to αIR3. Since clinically the TNBC are difficult to treat, the significant enhancement of low concentrations of EGCG on the cells response to αIR3 may be clinically very relevant. Particularly, we found that the response of the cells to IGF-I was not increased by EGCG despite the observed increase in levels of the receptor. As MDA-MB-231 cells produce a significant amount of endogenous IGF-II, we speculate that this amount of peptide could saturate the IGF-IR present on these cells and hence why addition of exogenous IGF-I has no further effect on cell proliferation. However, αIR3 would be able to compete with the endogenous IGF-II and to inhibit the cell growth but this mechanism remains to be confirmed.

We recently showed that IGFBP-2 is a novel positive regulator of the ERα and that this promotes cell survival in ERα-positive breast cancer cells (49). We confirmed in this study that the ability of EGCG to increase ERα was associated with an increase in IGFBP-2 and a reduction of ERα corresponded to a reduction of IGFBP-2. It will be interesting to investigate further the role of EGCG-induced changes of IGFBP-2 in breast cancer.

Having examined key molecules that have been implicated in regulating breast cancer cell growth and survival, we found no consistent changes that would explain the uniform inhibitory effects of EGCG. The ERα, Her2, and IGF-1R pathways contribute to different extents in the different cell lines that have varying phenotypes and some of the changes that we observed may have contributed to the effects of EGCG or they could have been compensatory responses.

Compared to *in vivo* conditions, cells *in vitro* are exposed to EGCG for very short time (only 48 h). We acknowledge that over this short period we have observed relatively small changes although significant, but presumably continuous long-term repeated exposure of cells *in vivo* to EGCG may have a more marked cumulative effect.

To promote safety and effectiveness of dietary reagents, derivatives with structural modifications such as pEGCG have been developed and synthesized. With changed structural characteristics, these phenolic compounds exert enhanced anti-proliferative effects in cancers (18). A nanoparticle-encapsulating EGCG has also been designed for oral administration in mice with human prostate cancer (50, 51). Our study highlights that the impact and specificity of EGCG in cells seems to be concentration-related and further studies investigating the effects of physiological levels of EGCG are essential.

## Conflict of Interest Statement

The authors declare that the research was conducted in the absence of any commercial or financial relationships that could be construed as a potential conflict of interest.

## Footnotes

^1^www.cancer.org
